# Case Report: Effective extracorporeal cardiopulmonary resuscitationin prolonged cardiac arrest

**DOI:** 10.3389/fcvm.2025.1643839

**Published:** 2025-10-28

**Authors:** Wangjun Yan, Guanxiu Tang, Hui Wang, Houshen Li, Luobin Liang, Yijiang Li, Shangping Zhao

**Affiliations:** ^1^ Department of Intensive Care Medicine, Hunan Aerospace Hospital, Changsha, China; ^2^ Department of Geriatrics, The Third Xiangya Hospital, Central South University, Changsha, China; ^3^ Guangdong Organ Support Engineering Technology Research Center, Shenzhen, China

**Keywords:** cardiac arrest, acute myocardial infarction, VA-ECMO, extracorporeal cardiopulmonary resuscitation, ECPR, case report

## Abstract

**Background:**

Cardiac arrest is a major cause of mortality, and outcomes after prolonged conventional cardiopulmonary resuscitation (CCPR) are often poor. Extracorporeal cardiopulmonary resuscitation (ECPR), involving veno-arterial extracorporeal membrane oxygenation (VA-ECMO), offers a rescue strategy for refractory cardiac arrest by providing cardiopulmonary support to allow treatment of underlying causes. This case report illustrates the potential of ECPR in an unusually prolonged cardiac arrest.

**Case description:**

We report a case of a 44-year-old man who experienced sudden prolonged cardiac arrest secondary to acute myocardial infarction and was successfully rescued with ECPR and VA-ECMO. The patient presented with 8 h of chest pain and went into ventricular fibrillation (VF) out of hospital; despite aggressive CCPR for approximately 155 min, return of spontaneous circulation was not achieved until ECPR was initiated. At 12:17 on the day of presentation, VA-ECMO was initiated in the emergency department after continuous CCPR. Subsequent coronary angiography revealed severe multivessel disease with critical proximal left anterior descending (LAD) artery stenosis, which was successfully treated with percutaneous coronary intervention (PCI) while the patient remained on ECMO support. The patient's post-arrest care included mechanical ventilation, targeted temperature management, inotropic and vasopressor support, antithrombotic therapy and aggressive neuroprotective measures. A large infarct in the left occipitotemporal lobe was identified on CT, but intensive care with hypothermia and sedation allowed the patient to survive with intact neurological function. ECMO was weaned off on day 9 of admission, and the patient was extubated on day 12. He subsequently made a full neurological recovery and was transferred out of the intensive care unit in stable condition.

**Conclusion:**

This case illustrates that even extremely prolonged cardiac arrest can result in good outcomes when managed promptly with ECPR and comprehensive critical care. It highlights the potential of ECPR to improve survival and neurological outcomes after refractory cardiac arrest and underscores the importance of rapid ECMO deployment in selected patients.

## Introduction

Cardiac arrest remains a leading cause of mortality worldwide, and survival after out-of-hospital or in-hospital cardiac arrest is generally poor (1, 2). Despite advances in basic and advanced life support, the prognosis after conventional cardiopulmonary resuscitation (CCPR) is often dismal, especially when resuscitation efforts must be prolonged. Extracorporeal cardiopulmonary resuscitation (ECPR), defined as the application of veno-arterial extracorporeal membrane oxygenation (VA-ECMO) during cardiac arrest, has emerged as a rescue therapy for patients with refractory cardiac arrest when return of spontaneous circulation cannot be achieved with CCPR (3, 4). By providing temporary cardiopulmonary support, ECPR restores organ perfusion and gas exchange, thereby “buying time” for the treatment of reversible causes such as myocardial infarction, massive pulmonary embolism, or drug overdose. International guidelines and expert consensus recommend considering ECPR after approximately 10–15 min of unsuccessful CCPR for selected patients in cardiac arrest, as time to cannulation has been correlated with neurological outcome (5, 6). Although robust randomized trials are lacking, observational studies and recent meta-analyses suggest that ECPR is associated with significantly improved survival and favorable neurological outcomes compared with CCPR alone (7–10).

Current registry data indicate that survival to discharge in ECPR-treated patients is on the order of 20%–30%, with most survivors achieving good neurological status (11–14). However, outcomes depend critically on patient selection, the timing of intervention, and post-resuscitation care (15). Patients with reversible acute coronary lesions or other treatable pathologies, and those receiving high-quality CPR, appear most likely to benefit (16, 17). Nonetheless, prolonged resuscitation is generally considered a poor prognostic sign, and cases surviving after very long “low-flow” intervals are rare. However, a recent case report described full recovery after 152 min of CPR supported by ECPR (18). These observations suggest that extended CPR is not universally futile if advanced therapies, such as ECMO, are applied in time.

In this report, we present the case of a middle-aged man with an out-of-hospital cardiac arrest of over 2.5 h duration who was treated with ECPR and PCI and ultimately survived with complete neurological recovery. We detail the patient's presentation, resuscitation timeline, and comprehensive critical care management. This case highlights both the challenges and potential of prolonged ECPR in cardiac arrest, and we discuss the lessons learned for clinical practice.

## Case description

A 44-year-old man with poorly controlled stage 3 hypertension for ten years presented with sudden severe chest pain on May 14, 2024. He had no history of diabetes, smoking, or previous surgeries. Around 7:00 AM, the patient experienced persistent, crushing substernal chest pain radiating to his arm. Approximately 2.5 h later, he became acutely short of breath and lost consciousness. Emergency medical services found him pulseless and in ventricular fibrillation (VF). Immediate bystander and paramedic efforts included defibrillation, intubation, and continuous chest compressions.

He was transported to Wangcheng District People's Hospital and arrived at 10:20 AM. On arrival, he remained comatose, apneic (intubated and ventilated), and in recurrent VF with absent pulse, gasping breaths, and dilated pupils. The initial 12-lead ECG showed ST-segment elevations suggestive of a large acute myocardial infarction involving the anterior and inferior walls. Immediate advanced cardiac life support (ACLS) was initiated, including repeated defibrillation, high-dose amiodarone and lidocaine and continuous mechanical chest compressions (Subang, Medsonic Henan). The patient was kept on a ventilator with FiO2 100% to ensure oxygenation during circulatory instability. Simultaneously, measures were taken to correct acidosis (*via* sodium bicarbonate) and maintain adequate perfusion with intravenous fluids.

Despite one hour of resuscitation, the patient remained in refractory cardiac arrest with no sustained pulse. At approximately 12:00 PM, the treating team contacted the regional cardiac center to prepare for ECPR. With the family's consent, veno-arterial ECMO cannulation (drainage cannula:16F, infusion cannula: 20F, Midos, Fresenius Beijing) was performed in the emergency department. At 12:17 PM, approximately 2 h and 35 min after the initial arrest, VA-ECMO flow was successfully established via femoro-femoral cannulation (Lifemotion, CBM Shenzhen).

Once on ECMO support, the patient's hemodynamics stabilized. He exhibited intermittent movement: he briefly opened his eyes to stimulation and withdrew limbs from painful stimuli. His electrocardiogram converted to sinus rhythm with a heart rate of approximately 100 beats per minute. On norepinephrine (2.0 μg/kg/min), the arterial pressure measured 108/58 mmHg. Oxygen saturation on the ventilator remained at 90%–98%. Pupils became equal (approximately 4.0 mm) with reactive light reflexes.

**Figure 1:**
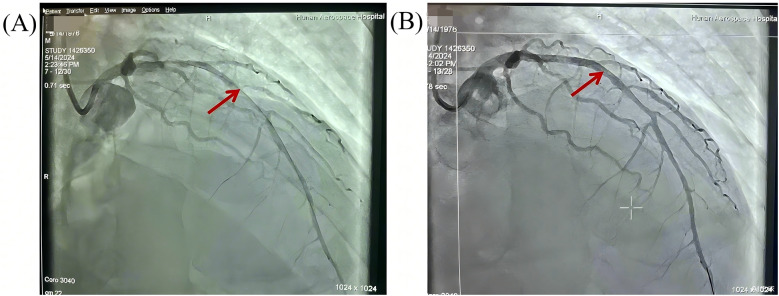
Pre- and post-PCI angiographic evaluation of diffuse stenosis in the left anterior descending artery. **(A)** Pre-PCI angiography: Diffuse tubular stenosis involving the proximal-to-mid LAD (arrows) with 50%–90% luminal narrowing. **(B)** Post-PCI angiography: a drug-eluting stent deployed across the lesion, achieving normalized antegrade flow (TIMI-3 flow).

Given the suspicion of acute myocardial infarction, the patient was emergently transferred to our tertiary cardiac center for definitive revascularization under ongoing ECMO support. He arrived in our catheterization laboratory at 1:50 PM. Coronary angiography revealed multivessel coronary artery disease, especailly diffuse tubular stenosis in the proximal-to-mid left anterior descending (LAD) artery, causing 50%–90% luminal narrowing (Figure 1). A drug-eluting stent was implanted to restore coronary perfusion, with a “door-to-balloon” time of 18 min.

After PCI, the patient was transferred to the intensive care unit (ICU) still on VA-ECMO and mechanical ventilation. On initial ICU assessment, he remained sedated and comatose (Glasgow Coma Scale = E2 Vt M4, with eyes opening to pain only and no verbal output). Pupils were 2.5 mm (left) and 2.0 mm (right) with sluggish light reflex. He was afebrile with heart rate 113/min on norepinephrine 2.0 μg/kg/min and dopamine 5 μg/kg/min; respiratory rate 15/min on ventilator assist; BP 75/44 mmHg with ECMO flow 2.5–3.0 L/min (target MAP−75 mmHg); SpO₂ 98%. General exam showed diffuse petechiae, mild cervical swelling at the internal jugular cannulation site with oozing, clear lung auscultation, and a distended, tense abdomen. ECMO cannulas were in place with mild swelling of the right thigh, no lower-extremity pulses detectable on that side. Neurologically, muscle tone was normal and plantar responses were flexor.

Based on these findings, the admission diagnoses were: (1) acute extensive anterior myocardial infarction (Killip class IV) with cardiogenic shock; (2) ventricular fibrillation cardiac arrest; (3) post-CPR refractory cardiac arrest on VA-ECMO; (4) post-resuscitation hypoxic-ischemic encephalopathy; (5) possible intestinal ischemia or infection; and (6) hypertension (Stage 3, very high risk).

## Management and outcomes

### Cardiovascular support

**Figure 2:**
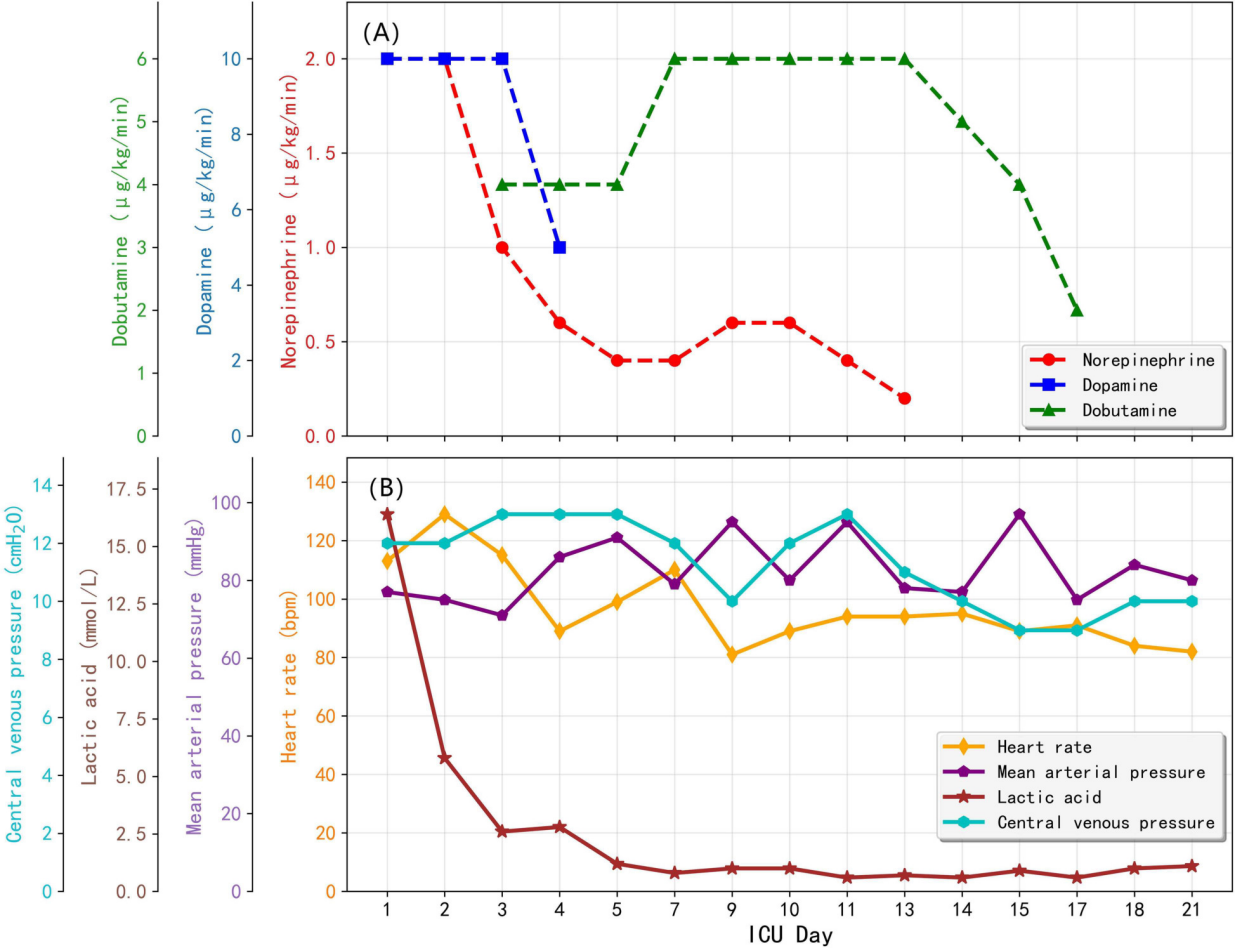
Changes of vasopressor doses and corresponding hemodynamic parameters over ICU stay. **(A)** Vasopressor doses; **(B)** Hemodynamic parameters.

VA-ECMO provided full circulatory support from Day 1 through Day 10. Initial ECMO flows were maintained at 2.5–3.0 L/min, targeting a mean arterial pressure (MAP) of −75 mmHg. Norepinephrine (2.0 μg/kg/min) and dopamine (5 μg/kg/min) were used post-admission with vasoactive support radually reduced over the first 2–3 days. Amiodarone was continued to manage ventricular arrhythmias. Serial transthoracic echocardiography showed progressive myocardial recovery: left ventricular ejection fraction (LVEF) rose from 25% on Day 1%–36% by Day 3, and further to 58%–65% by Day 8–12. ECMO was successfully weaned and discontinued on Day 10 due to improved ventricular function. Changes of vasopressor doses and corresponding hemodynamic parameters during ICU was shown in Figure 2. Right leg peripheral perfusion was monitored with no major ischemia noted.

### Anticoagulation management

A continuous heparin infusion was initiated, targeting an activated clotting time of −180–200 s. D-dimer and antithrombin III levels guided therapy. On Day 2, the ECMO oxygenator membrane was exchanged due to hypercoagulability concerns. Subsequent anticoagulation transitioned to low-molecular-weight heparin (enoxaparin 0.2 mg/kg q12 h), and heparin was discontinued by Day 4 due to heightened bleeding risk. Post-decannulation, therapeutic anticoagulation was stopped, and the patient was managed on low-dose aspirin and clopidogrel for the stent.

### Respiratory and neurological support

The patient remained intubated and mechanically ventilated throughout ECMO support. Targeted temperature management (TTM) was employed for neuroprotection. Cooling was started on ICU admission: a cooling cap (ice cap) was applied and the ECMO heat exchanger set to maintain mild hypothermia (33–34 °C) for approximately 48–72 h. Sedation was deepened during TTM with midazolam and sufentanil to achieve burst suppression on EEG. Additionally, to control high intracranial pressure surges, a sedative “barbiturate coma” regimen was used, combining chlorpromazine, promethazine, and pethidine (meperidine) on Day 2 when the patient spiked a high fever (39 °C). Hyperosmolar therapy was also employed: mannitol 125 ml q8 h and hypertonic saline (10% NaCl) were given to maintain serum sodium ∼150–155 mmol/L. Dexamethasone 10 mg q12 h and albumin 10 g q12 h were administered to stabilize the blood-brain barrier. Continuous cerebral oxygen saturation monitoring showed left-side rSO₂ around 65%–80% and right-side around 72%–79% over days 2–5; a transient drop (left 65%, right 55%) on Day 2 triggered immediate imaging.

### Neurological course

**Figure 3:**
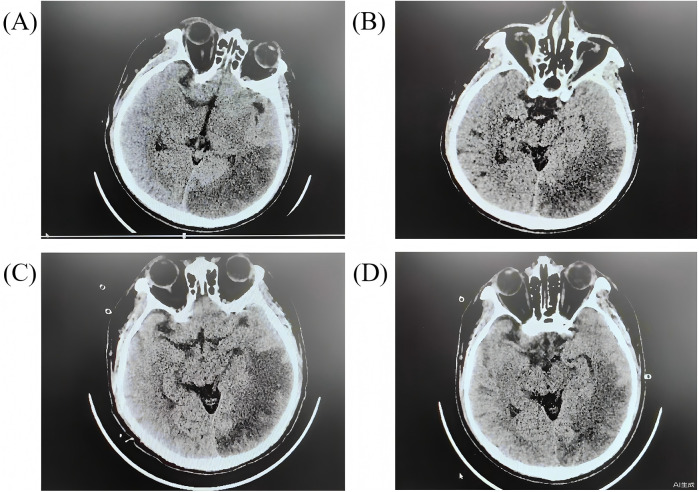
The head CT scans for the patients during hospitalization. **(A)** day 2 after arrest; **(B)** day 7 after arrest; **(C)** day 11 after arrest; **(D)** day 15 after arrest.

On Day 2, a right pupil 2.5 mm vs. a left pupil 3.5 mm (sluggish on the left) was noted. A stat non-contrast head CT revealed a large acute infarction in the left occipital-temporal (Figure 3A). Neuroprotective medications, including edaravone and butylphthalide, were added. On Day 3, the patient developed signs of central diabetes insipidus. Desmopressin (pitressin) was given, and mannitol was discontinued to avoid exacerbating hypernatremia. Hypothermia was discontinued on Day 4, and the patient was gradually rewarmed to normothermia over the next 24–48 h. By Day 5, sedation was lightened, and attempts at neurologic assessment were made. Remarkably, on Day 7 the patient showed purposeful movements and coherent speech with minimal sedation. Over the next days his orientation improved. Neurological exams eventually normalized with no hemiparesis.

### Other support and complications

**Figure 4:**
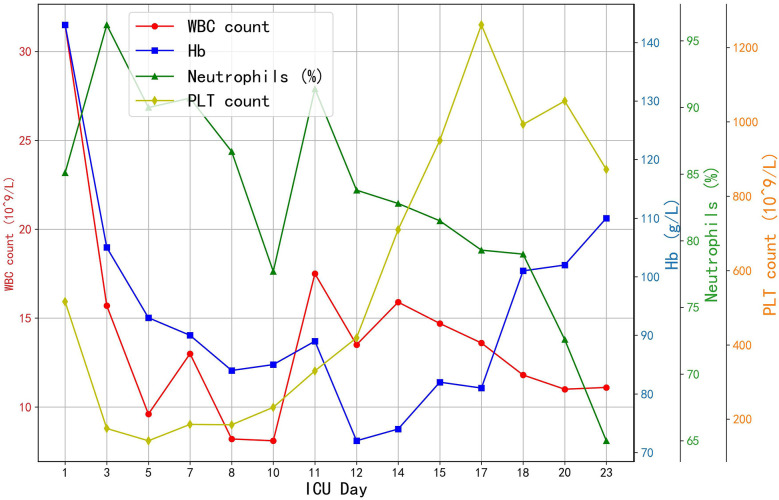
The trends of hematological parameters. WBC, white blood cell count; Hb, haemoglobin; PLT, platelet count.

Prophylactic broad-spectrum antibiotics were administered due to high risk of infection. The patient received meropenem (1 g q8 h from Day 1) and later polymyxin B (from Day 7) to cover resistant organisms suggested by cultures. The trends of hematological parameters during hospitalizations are showed in Figure 4. A brief course of colistin was also used. Daily chest x-rays and lab tests showed improving inflammation. Gastrointestinal mucosal protection was provided with proton-pump inhibitors and sucralfate. Enteral nutrition was initiated on Day 3. Hepatic support measures (glutathione and magnesium isoglycyrrhizinate infusions) were given to mitigate liver injury from shock. An “Si-Mo-Tang” herbal decoction and lactulose were used to keep intestinal motility. Acute kidney injury did not develop, and no dialysis was required. Coagulation panels were monitored frequently; during ECMO there was mild thrombocytopenia (nadir platelets −80,000/µl), managed with platelet transfusions and eventual platelet apheresis on Day 4 to lower circuit clot risk. No major bleeding events occurred.

### Rehabilitation and follow-up

By Day 7, the patient was awake and breathing spontaneously. The ventilator was gradually weaned, and he was successfully extubated to high-flow nasal oxygen on Day 11. Early rehabilitation was begun in ICU: bedside sitting, passive limb exercises, and then active sitting out-of-bed as tolerated. He underwent a tracheostomy on Day 9, which was removed at extubation. With improving cardiac function and no other organ failures, ECMO decannulation proceeded on Day 10. His ECG changes resolved, and serum troponin levels trended downward; kidney and liver function tests also normalized. On Day 15, repeat head CT scans confirmed the stable stroke without hemorrhagic conversion (Figure 3D). He maintained normal serum neuron-specific enolase and S100B levels in the second week, supporting a good prognosis. The patient was transferred from the ICU to the general cardiac ward 21 days after cardiac arrest. He continued inpatient rehabilitation and was discharged on Day 36 with no motor or cognitive deficits. At 3-month follow-up, he had returned to all prior activities with no dyspnoea or neurological complaints.

## Discussion

### Importance of rapid ECPR

ECPR was the turning point in this patient's resuscitation. Once on VA-ECMO, organ perfusion was restored despite the failure of conventional CPR. The ELSO consensus emphasizes that ECPR is indicated for patients with cardiac arrest refractory to CCPR, especially when a reversible cause is suspected (19, 20). In this case, the cause was an acute MI amenable to PCI, performed under ECMO stabilization. Timely identification of the treatable lesion and prompt revascularization were critical. Retrospective data suggest CPR improves outcomes in cardiac arrests of presumed cardiac origin with reversible pathology. In contrast, it's less beneficial for irreversible or non-cardiac causes (21). The operational success here also related to the rapid ECMO initiation. In prolonged low-flow cardiac arrest, minimizing procedural delays is key. In emergent ECPR, where neurologic and survival outcomes are time-sensitive, equipment optimized for ease of use and rapid deployment is essential. This case highlights the pivotal role of early reversible causes identification and rapid circulation restoration in managing refractory cardiac arrest.

Guidelines recommend considering ECPR after 10–15 min of ineffective CPR (18). Though the “low-flow” time before ECMO in this case far exceeded typical recommendations, success likely due to high-quality CPR and the patient's relatively young age with minimal comorbidities. The cardiac arrest was considered witnessed, with paramedics arriving soon after collapse and initially presented with VF, a shockable rhythm linked to a better prognosis (22). These factors match the ECPR candidacy criteria. Although prolonged resuscitation is generally associated with mortality of over 95%, ECMO has shown good outcomes in some cases. Retrospective analyses emphasize the significance of high-quality CPR and minimal low-flow time. For instance, a JAAM-OHCA ECPR registry study showed that shorter hospital-level door-to-ECPR times are associated with improved outcomes in OHCA patients and could serve as a quality indicator for ECPR processes across hospitals (23). Our patient's case is consistent with these findings, as he was a young patient with a reversible cause, received continuous compressions, and had timely ECMO initiation.

Recent literature increasingly supports aggressive resuscitation strategies even after prolonged CPR durations. For example, a case report from the Department of Critical Care Medicine, Nantong Third People's Hospital (Nantong, China) described a patient who underwent 152 min of CPR and was rescued using ECPR together with continuous renal replacement therapy (CRRT), eventually achieving full neurological recovery (18). This remarkable outcome challenges the long-standing notion that CPR beyond one hour is invariably futile. While prolonged CPR is typically associated with dismal outcomes, the advent of advanced extracorporeal support technologies appears to extend the “salvage window” (3). In support of this evolving view, the recent meta-analysis published in Critical Care (2024) analyzed randomized and observational studies comparing ECPR and CCPR across both in-hospital and out-of-hospital cardiac arrest settings. Their updated analyses reaffirmed that ECPR significantly reduces in-hospital mortality and improves short-term neurological outcomes and 30-day survival relative to conventional CPR (8). ECPR programs now report survival rates around 30% for in-hospital arrests and 10%–20% for out-of-hospital arrests, with over 85% of survivors having favorable cerebral performance (12). While critics argue that prolonged resuscitations waste resources, our patient's favorable outcome suggests that select cases may justify the effort. Future research should better define which patients will benefit from extended ECPR.

### Interventions and supportive care

Several aspects of the patient's management likely contributed to recovery. Post-arrest, therapeutic hypothermia and deep sedation were used to mitigate reperfusion injury. As observational data suggests that maintaining core temperature at 33–36 °C for 24–48 h improve neurological recovery after prolonged arrest (24), hypothermia was continued for 72 h in our patient due to extended downtime. We also used osmotherapy and steroids to control cerebral edema, along with continuous rSO₂ monitoring to guide cerebral perfusion. Neuroprotective agents (edaravone and butylphthalide) were added when a large infarct was revealed on CT. While the benefit of each intervention can't be quantified, their combined effect likely supported brain recovery. Circulatory support was optimized throughout. Inotropes and vasopressors were adjusted to maintain perfusion, and serial echocardiography guided ECMO weaning as ventricular function improved. The gradual improvement in LVEF indicates myocardial stunning recovered over time, with ECMO providing a bridge during this period. Coronary revascularization likely limited infarct size by achieving reperfusion within one hour of ECMO cannulation. Antithrombotic therapy (aspirin, P2Y₁₂ inhibitor, heparin) was carefully balanced to prevent stent thrombosis without causing hemorrhage, with no major bleeding or stroke hemorrhagic conversion occurring. Given the patient's prolonged ICU stay and invasive devices (ventilator, ECMO cannulas), critical infection prevention was necessary. Broad-spectrum antibiotics were used empirically, adjusted based on cultures and procalcitonin trends. The patient did not develop sepsis or multi-organ failure, which likely aided recovery.

### Complications: brain infarction

The case was complicated by a large ischemic stroke (Figure 3). Neurological injury is the most feared complication of cardiac arrest and ECMO (25). It is not possible to determine whether the stroke occurred during the arrest or during the peri-ECMO period. Fortunately, the infarct was in the left occipitotemporal region, largely non-motor, so that no hemiparesis ensued. The patient's intact neurologic status allowed aggressive therapy to continue. Whether an ECPR patient has an infarct or hemorrhage can be the key determinant of outcome. In this case, early detection and treatment of the infarct (and the absence of hemorrhage) were beneficial. Given his diabetes insipidus and imaging findings, we monitored endocrinologic and metabolic parameters closely. The transient diabetes insipidus was likely central and was promptly treated with desmopressin to prevent hypernatremia and further neural damage. Other organ functions (renal, hepatic) remained stable, which may reflect the protective effect of ECMO perfusion (26).

A key lesson is that CPR duration alone should not be an absolute contraindication to ECPR when other favorable factors exist. Although many centers question initiating ECMO after hours of failed CPR, emerging evidence, including a 152 min case, indicates exceptional survival chances (18, 23). Our success may encourage other centers to consider ECPR even in “prolonged” arrests, provided logistic support and post-resuscitation care are in place. Rapid coordination among pre-hospital, emergency, and cardiac ICU teams was crucial. This case also underscores the need for public education on CPR and dispatcher-assisted defibrillation, as early efforts likely delayed irreversible injury and made ECPR possible. However, as this report describes a single patient, no bias adjustment or confounder control was possible; this limitation underscores that our findings are descriptive and hypothesis-generating rather than inferential. Without a comparator group, causality between ECPR and outcome cannot be inferred; this case is presented as an illustrative example to stimulate further investigation. Development of predictive models or decision algorithms for ECPR candidacy, using large epidemiological datasets, will be essential to guide clinical decision-making.

For clinicians, this case reinforces several key points (27). Firstly, activate the ECMO team early in refractory cardiac arrest, ideally within 60 min, but consider it even later if the patient can tolerate prolonged CPR. Secondly, in cardiac arrests due to myocardial infarction, aggressive revascularization under ECMO support can restore circulation and improve survival. Moreover, comprehensive post-arrest management is essential for brain function salvage after prolonged arrest. Lastly, successful ECPR requires a well-practiced team and protocols spanning multidisciplinary teams.

## Conclusion

This case highlights that even extremely prolonged resuscitations may be compatible with good outcomes when managed with rapid ECPR and comprehensive critical care. It supports the growing evidence that ECPR can save lives in refractory cardiac arrest and should be considered in carefully selected patients.

## Data Availability

The raw data supporting the conclusions of this article will be made available by the authors, without undue reservation.
